# Vegetation and floristics of a lowland tropical rainforest in northeast Australia

**DOI:** 10.3897/BDJ.4.e7599

**Published:** 2016-02-24

**Authors:** David Y. P. Tng, Deborah M. G. Apgaua, Mason J Campbell, Casey J Cox, Darren M Crayn, Françoise Y Ishida, Melinda J Laidlaw, Michael J Liddell, Michael Seager, Susan G. W. Laurance

**Affiliations:** ‡ Centre for Tropical, Environmental, and Sustainability Sciences, College of Marine and Environmental Sciences, James Cook University, Cairns, Australia; § Departamento de Ciências Florestais, Universidade Federal de Lavras, Lavras, Brazil; | Australian Tropical Herbarium, Cairns, Australia; ¶ Department of Science, Information Technology, Innovation and the Arts,Queensland Herbarium, Brisbane, Australia

**Keywords:** Australia, lianas, permanent plot, rain forest, shrubs, tropical rain forest

## Abstract

**Background:**

Full floristic data, tree demography, and biomass estimates incorporating non-tree lifeforms are seldom collected and reported for forest plots in the tropics. Established research stations serve as important repositories of such biodiversity and ecological data. With a canopy crane setup within a tropical lowland rainforest estate, the 42-ha Daintree Rainforest Observatory (DRO) in Cape Tribulation, northern Australia is a research facility of international significance. We obtained an estimate of the vascular plant species richness for the site, by surveying all vascular plant species from various mature-phase, remnant and open vegetation patches within the site. We also integrate and report the demography and basal areas of trees ≥ 10 cm diameter at breast height (dbh) in a new 1-ha core plot, an extension to the pre-existing forest 1-ha plot under the canopy crane. In addition, we report for the canopy crane plot new demography and basal areas for smaller-size shrubs and treelets subsampled from nine 20 m^2^ quadrats, and liana basal area and abundance from the whole plot. The DRO site has an estimated total vascular plant species richness of 441 species, of which 172 species (39%) are endemic to Australia, and 4 species are endemics to the Daintree region. The 2 x 1-ha plots contains a total of 262 vascular plant species of which 116 (1531 individuals) are tree species ≥ 10 cm dbh. We estimate a stem basal area of 34.9 m^2^ ha^-1^, of which small stems (tree saplings and shrubs <10cm dbh) and lianas collectively contribute c.4.2%. Comparing the stem density-diversity patterns of the DRO forest with other tropical rainforests globally, our meta-analysis shows that DRO forests has a comparatively high stem density and moderate species diversity, due to the influence of cyclones. These data will provide an important foundation for ecological and conservation studies in lowland tropical forest.

**New information:**

We present a floristic checklist, a lifeform breakdown, and demography data from two 1-ha rainforest plots from a lowland tropical rainforest study site. We also present a meta-analysis of stem densities and species diversity from comparable-sized plots across the tropics.

## Introduction

Tropical rainforests are globally recognized for their rich biodiversity, socio-economic importance, the ecosystems services they provide, and their potential for buffering the impacts of climate change. In terms of ameliorating the effects of climate change, tropical rainforest represent some of the most carbon-dense terrestrial ecosystems on the planet, and play a key role in global carbon cycling (Clark 2002). The study of tropical rainforest biodiversity has therefore been of immense interest to biologists, and recently also to those with an interest in carbon accounting (Sierra et al. 2007; Liddell et al. 2007; Murphy et al. 2013).

While many methods exist for measuring diversity (Gordon and Newton 2006), plot-based methods remain the most widely-used (Campbell 1994). Yet, despite the long tradition of plot-based studies, most studies still focus on trees, and retain a subjective diameter cutoff of ≥ 10 cm at breast height (Phillips et al. 2003). However, the often overlooked non-tree components of rainforests are deserving of attention as numerous studies have documented the significant contribution of these lifeforms to species richness and ecosystem processes (Richards et al. 1940; Gentry and Dodson 1987; Ewel and Bigelow 1996; Poulsen 1996). Likewise for aboveground biomass, the exclusion of smaller trees and shrubs and herbaceous lifeforms can lead to underestimated values of carbon stocks (Preece et al. 2012). In addition, the ability to track the dynamics of a rainforest community is limited if one only works on mature trees as has been widely recognised in the establishment of the Centre for Tropical Field Studies Large Plot Network where in the standard methodology (Condit 1998) stems are measured down to 1 cm.

The Wet Tropics bioregion covers an area of approximately 2 million ha spaning some 450 km from 15^o^40'S to 19^o^15'S along the tropical east coast of northern Australia. This bioregion encompasses a mosaic of terrestrial ecosystems ranging from sclerophyll vegetation, seasonally dry tropical forest, tropical wet forest and wetlands (Goosem et al. 1999; Kemp et al. 2007). Among the different rainforest types present in the region, coastal lowland rainforest has been identified as being endangered, on the basis of IUCN guidelines (Metcalfe and Lawson 2015). Globally, lowland tropical rainforest is increasingly recognized as a forest ecosystem particularly vulnerable to the projected climate change impacts such as extended drought and warming (Van Nieuwstadt and Sheil 2005; Phillips et al. 2009; Corlett 2011). Long-term vegetation plot and floristic composition data are therefore important baselines for monitoring and understanding ecological processes of such forests.

In the Daintree region near the northern end of the Wet Tropics, coastal lowland rainforest is still well preserved and contiguous with a 17,000 ha forest tract within the Daintree National Park. The lowland rainforests in the Daintree region, particularly those near Cape Tribulation have been considered by some workers to represent the ‘optimal development of rainforest in Australia’ (Tracey 1982), underpinning the importance for long-term monitoring of such sites. In 1998, the Australian Canopy Crane Research Station was established (now known as the Daintree Rainforest Observatory), and a Leibherr 91EC industrial T-crane was installed on the site to facilitate canopy access by researchers (Stork and Cermak 2003; Stork 2007). The Daintree Rainforest Observatory (DRO) focal monitoring plot (crane plot) was initially set up as a circular 0.95 ha plot assessable by a canopy crane (Laidlaw et al. 2007). For the first 10 years of its lifetime this was the only canopy crane in Australia, placing the facility of considerable national and international value, among a global network of canopy crane sites (Stork 2007; Nadkarni et al. 2011). Although only 1-ha, the crane plot represents the largest of the long term continuously-monitored coastal lowland tropical rainforest permanent monitoring plots in Australia, CSIRO having monitored a set of smaller 0.5-ha plots since 1971 (Bradford et al. 2014). Since its inception, the DRO has been a hotspot of scientific activity and has been utilized as a study site either in its entirety (e.g. Boulter et al. 2005; Liddell et al. 2007; Kitching et al. 2007; Apgaua et al. 2015; Nolf et al. 2015), or as part of regional (Falster and Westoby 2005), continental (Ziemińska et al. 2015) or international studies (e.g. Rosell et al. 2013; Olson et al. 2014; Atkin et al. 2015)

As part of a program of development of the DRO to better facilitate research and educational activities, we set up an additional 1-ha monitoring plot (henceforth core plot), conducted demographic assessments of nontree life-forms from subsampled quadrats within the crane plot, and undertook a floristic survey of the entire research site to obtain an estimate of the overall floristic richness of the site. As a result of those activities, we provide here: (i) an updated analysis and synthesis of the vascular plant structure of the now 2 x 1-ha DRO plots; (ii) demographic data of the non-tree lifeforms from the crane plot, and; (iii) an analysis of the phylogenetic structure of the flora of these plots and the site. The data will form a new baseline description of the stand structure and diversity of the vegetation of the research station that will facilitate and encourage long-term ecological research, and enable comparative studies with similar plots worldwide. The core 1-ha plot has been established for the Cape Tribulation/Daintree node of the Far North Queensland SuperSite, to place the DRO inside a national plot monitoring network, the TERN SuperSite network (http://www.tern-supersites.net.au) (Karan et al, *in prep.*).

## Sampling methods

### Study extent

**Figure 1.**
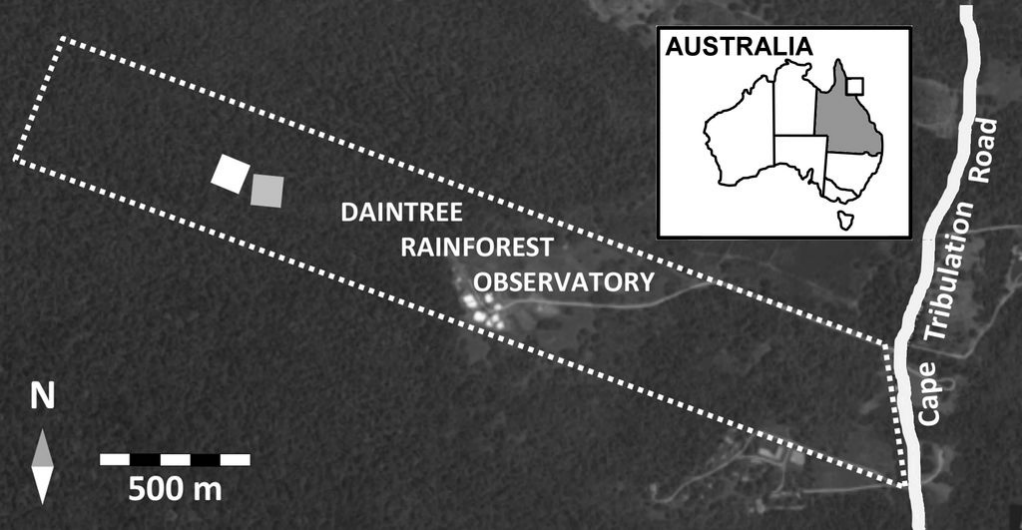
Aerial image of the Daintree Rainforest Observatory, Cape Tribulation. The white dotted line indicates the boundaries of the property. The grey and white boxes within the property denote the location of the crane and new core 1-ha plot respectively.

The Daintree Rainforest Observatory (DRO) is a 42-ha property owned by the James Cook University, and is located at Cape Tribulation (16°06′S, 145°26′E, c. 50 m elevation), north-eastern Queensland, Australia (Fig. 1). It is situated within a semi-enclosed coastal basin formed by ridges running east-west to an upland massif (Grove et al. 2000). Soils at the site are acidic and comprised of strongly weathered brown dermosols (Australian Soil Classification: Isbell 2002). The climate of the site is tropical, with a mean annual temperature of 24.4^o^C. The site receives a mean annual rainfall of approximately 5100 mm (2006 - 2014), with a distinct 3 month dry season (August-October: monthly rainfall <100mm) and 4 month wet season (Jan-Apr, monthly rainfall >500mm) (Bureau of Meteorology 2015). The site also experiences tropical cyclones, the most recent in 2014 (Bureau of Meteorology 2015).

The vegetation within the DRO consists primarily of mature coastal lowland tropical rainforest. Regionally, this rainforest vegetation is described as Complex Mesophyll Vine Forest (Tracey 1982, Goosem et al. 1999). The remaining vegetation at the site consists of edge vegetation associated with the mature rainforest, secondary forests remnants of varying sizes and ages, revegetation plantings, anthropogenic grasslands, and urban clearings.

### Sampling description

Within the mature rainforest estate there are two 1-ha plots located 20 m apart from each other and separated by a creek in which every tree ≥ 10 cm diameter at breast height (dbh) has been measured, tagged, and identified to species. The original circular 0.95-ha crane plot over-towered by the canopy crane was first surveyed in 2000 (Laidlaw et al. 2007), and has since been recensused four times, most recently in 2015. In 2010, extra trees were tagged and measured to extend the plot boundaries into a square 1-ha plot. The core 1-ha plot was initially set up in 2001 (Laidlaw et al. 2007) but reestablished in 2013, and recensused in early 2015. Tree diameter measurements were taken at 1.30 m from the ground on the uphill side of the bole, or immediately above buttressed roots or other stem abnormalities. Each tree was marked with a painted line to indicate the location of dbh measurement (point of measure). Coppiced stems ≥ 5 cm in diameter have been recorded as second stems of the same individual tree.

Non-tree lifeforms were subsampled within the crane plot. All individual tree saplings (≥1cm dbh) and shrubs within nine randomly assigned 10 m x 2 m quadrats were tagged and measured. For our purposes, shrubs were defined as species that do not or rarely exceed 6 m in height, and all shrub individuals ≥ 0.4 cm diameter 5 cm above ground level were marked and measured. We deviated from the more typical protocol of measuring stems ≥ 1 cm or ≥ 2.5 cm (e.g. Gentry and Dodson 1987; Leigh 1999, p 200-202) to obtain a more complete census of shrubs.

All lianas (≥ 1 cm diameter) were marked and measured in the crane plot using standard protocols (Gerwing et al. 2006, Schnitzer et al. 2008). As an exception, we did not calculate the basal area and/or stem abundance of climbing palms or/ rattans (*Calamus* spp.) as their clonal stems and continuous growth form render estimations of abundance and biomass impractical. However we obtained total liana cover and cover per liana species (including *Calamus* spp. as a group) as a percentage of tree canopy area for 58 random tree canopies. For this cover estimate, we used the mean of values visually estimated by two independent observers. In order to circumvent potential bias in cover estimates, both observers were unaware of the number, basal area and biomass of lianas recorded for each host tree as recorded in the understorey survey. Liana cover estimates included the sunlit and tree canopy areas that were visible directly overhead from the crane gondola (Cox et al, *in review*).

Finally, to obtain an overall estimate of the vascular plant species richness, we surveyed all vascular plant species within the site using an *ad hoc* approach (e.g. Gordon and Newton 2006). Species nomenclature follows Bostock and Holland (2013). Voucher specimens for all species collected were lodged either in the Daintree Rainforest Observatory reference herbarium or at the Australian Tropical Herbarium (CNS).

***Analysis***

We calculated a number of commonly used diversity indices for the tree stems ≥ 10 cm dbh within the combined 2 x 1-ha plot, and separately for the crane and core plots. These include the Margalef's species richness index, Shannon diversity index, Fisher’s alpha, and Pielou's and Simpson’s evenness index. Diversity indices were calculated using PAST 3.02a software (Hammer et al. 2001).

## Geographic coverage

### Description

The Daintree Rainforest Observatory (DRO) is a 42-ha property owned by the James Cook University, and is located in Cape Tribulation at approximately 50 m elevation, north-eastern Queensland, Australia (Fig. 1).

### Coordinates

16°06′S and Latitude; and 145°26′E Longitude.

## Taxonomic coverage

### Description

*Floristics and stand structure*

**Figure 2.**
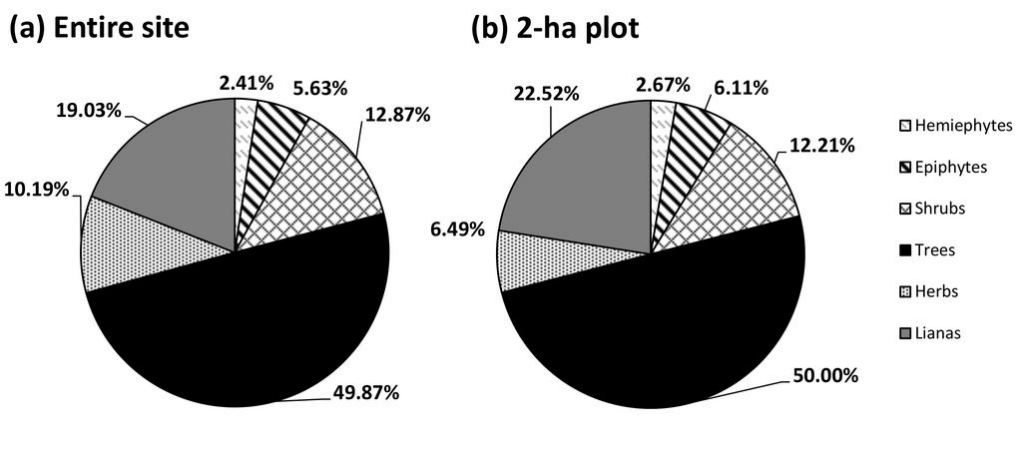
Native vascular plant lifeform distribution of (a) the entire Daintree Rainforest Observatory site (n = 373 spp.) and (b) the combined 2 x 1-ha permanent plots (n = 267 spp.).

The vascular plant survey of the Daintree Rainforest Observatory site documented 441 species (385 native, 56 non-native) representing 307 genera and 115 families. Of these, 172 species (39%) are endemic to Australia (Suppl. material 1). The distribution of lifeforms in descending order of diversity are as follows: trees (49.9% of species), lianas (19%) herbs and graminoids (10.2%), shrubs (12.9%), epiphytes and mistletoes (5.6%) and hemiepiphytes (2.4%) (Fig. 2).

**Figure 3.**
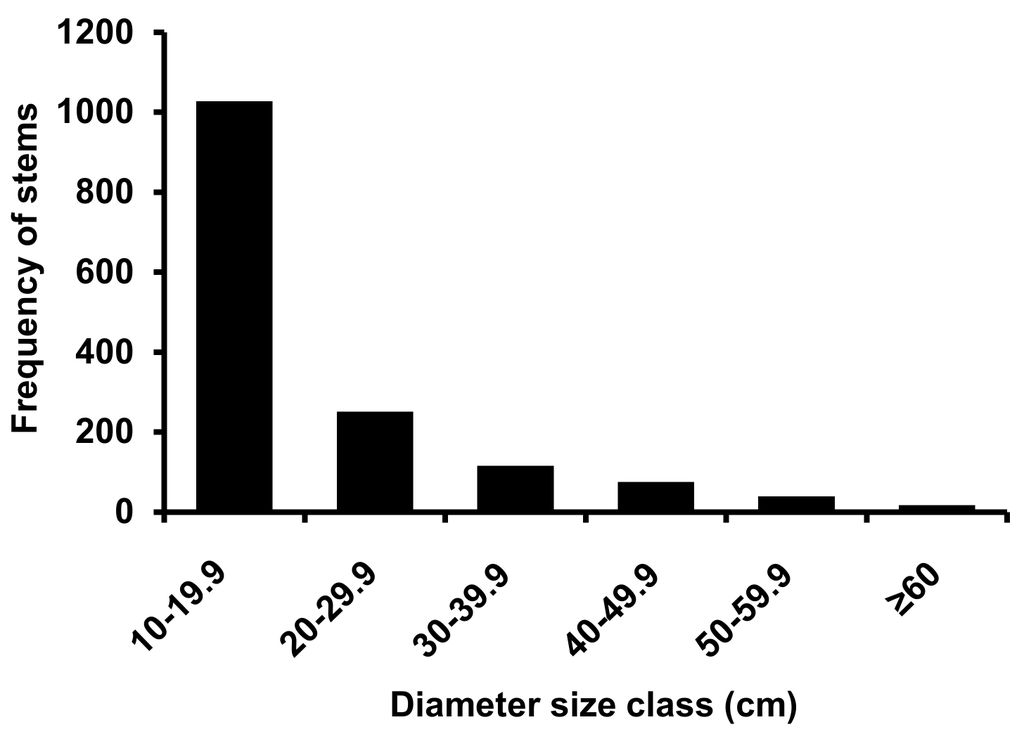
Diameter size classes of tree individuals (> 10 cm dbh) from the combined 2 x 1-ha Daintree Rainforest Observatory plots.

Within the 2 x 1-ha plots, we recorded 268 native species and one non-native (the fern *Pityrogramma calomelanos*) representing 202 genera and 87 families (Suppl. material 1). The lifeform distributions are similar to that of the entire site with the exception of a smaller percentage of herbs and graminoids (Fig. 2). We counted 1531 tree stems ≥ 10cm dbh, consisting of 115 species from 87 genera and 46 families (Table 1). In terms of family importance values, the Lauraceae, Arecaceae and Proteaceae were the top three most important (Table 2), comprising 13.1, 18.6 and 6.6% of the total stems respectively. The most common species were the subcanopy trees *Cleistanthus myrianthus* (Phyllanthaceae), *Normanbya normanbyi* (Arecaceae) and *Licuala ramsayi* (Arecaceae) comprising 9.4, 9.4 and 7.7% of the total stems respectively. Most of these stems fell within the 10-30 cm dbh size classes (Fig. 3). Thirty-one species were represented by a single stem. The estimated total basal area contributed by stems ≥ 10cm dbh is 33.4 m^2^ ha^‑1^ and the ten species in the plot with the highest importance value index constituted 55.3% of the basal area, of which the three top contributors are the canopy trees *Cardwellia sublimis* (Proteaceae), *Endiandra microneura* (Lauraceae) and the subcanopy tree *Cleistanthus myrianthus* (Phyllanthaceae) (Table 3).

A total of 71 stems of tree saplings (27 species) and 154 shrubs (12 species) were measured in the nine 20 m^2^ subplots in the crane plot, which extrapolates to 394 tree sapling and 856 shrub stems per ha. The tree saplings represent a subset of the tree species already in the ≥ 10 cm dbh size range, with the exception of one individual from one species (*Beilschmiedia bancroftii*). Among the tree saplings *Cleistanthus myrianthus* was the most abundant tree sapling, comprising 22.5% of the stems and 9.6% of the basal area of all tree saplings, followed by *Endiandra microneura* (15.5% of stems, 14.7% basal area) (Table 4). In terms of shrubs, *Haplostichanthus ramiflorus* (Annonaceae) was the most abundant and comprised over 52% of the stems and 71.9% of the shrub basal area). This was followed by *Atractocarpus hirtus* (Rubiaceae) (16.4% of stems, 8% shrub basal area) and *Bowenia spectabilis* (Zamiaceae) (15.1% of stems, 3.1% shrub basal area) (Table 5). Collectively, the basal area of tree saplings and shrubs extrapolates to 0.54 m^2^ ha^-1^, and represents an estimated 1.5% of the total basal area for the combined 2 x 1-ha plot.

We recorded 1072 liana stems (> 1 cm dbh) with an estimated basal area of 0.924 m^2^ ha^-1^, which represented 2.7% of the total basal area for the 2 x 1-ha plots. Of the 58 canopy trees examined for liana load, 19 species of lianas (32% of all liana species documented from the crane plot) were recorded. In particular, *Merremia peltata* (Convolvulaceae), *Entada phaseoloides* (Fabaceae) and *Tetrastigma nitens* (Vitaceae) were present on a large percentage of the sampled trees (84, 52 and 43% host trees respectively), and exhibited high mean covers (24.5, 8.4 and 4.1% respectively).

*Endemism and biogeography*

Endemism was primarily represented at the species level and by trees, with 168 species (45% of native species) endemic to Australia, and among the tree stems ≥ 10cm dbh in the 2 x 1-ha plots, 63 (54.7%) species are endemic to Australia. These patterns are largely attributable to tree species within the families Lauraceae, Myrtaceae, Proteaceae and Sapindaceae (Table 3, Suppl. material 1). Some other species such as *Ryparosa kurrangii* (Achariaceae), are restricted to the Daintree region within Australia, but found in tropical forests in the Asian Pacific. In addition, the lowland rainforest plots harbor a number of endemic species restricted in distribution to the Daintree region, including *Cupaniopsis diploglottoides* (Sapindaceae), *Endiandra grayi*, *E. microneura* (Lauraceae), *Haplostichanthus ramiflorus* (Annonaceae) and *Normanbya normanbyi* (Arecaceae).

## Usage rights

### Use license

Creative Commons CCZero

### IP rights notes

This dataset can be freely used, provided it is cited.

## Data resources

### Data package title

Table A1. Daintree Rainforest Observatory vascular plant species list and stem abundances (≥ 10 cm dbh) within the 2 x 1-ha monitoring plots

### Number of data sets

1

### Data set 1.

#### Data set name

Table A1. Daintree Rainforest Observatory vascular plant species list and stem abundances (≥ 10 cm dbh) within the 2 x 1-ha monitoring plots

#### Number of columns

5

#### 

**Data set 1.** 

Column label	Column description
Species	Species names
Family	Botanical family
Lifeform	Lifeform
Status	Exotic, native or endemic status in Australia
Plot Presence or Abundance	Plot Presence or Abundance

## Additional information

### Discussion

We synthesize and report the floristics and vegetation structure of tropical lowland rainforest within the Daintree Rainforest Observatory. Our synthesis takes into account the contribution of less commonly studied lifeforms (e.g. shrubs and lianas) to biodiversity and woody basal area for future plot comparisons.

Lifeform composition studies are generally uncommon for tropical rainforests, but Ewel and Bigelow (1996) have provided a global synthesis on which we can base comparisons. The contribution of the tree lifeform to the DRO site and 2 x 1-ha plots is higher than Neotropical forests by an order of magnitude (Ewel and Bigelow 1996), and comparable with figures reported by Stocker (1988) for other Australian wet tropics rainforests. The percentage of lianas is comparable to Neotropical forests, but the contribution of shrubs and epiphytes however, is relatively low. Relative to Neotropical sites, the lower epiphyte composition is attributable to the more seasonal climate in the Australian lowland tropics, and also the lack of some specialist epiphyte lineages (i.e. Bromeliaceae) to fill the epiphytic niche (Gentry and Dodson 1987).

The full floristics within the 2 x 1-ha plots encompasses most of the primary rainforest species within the DRO site, and the remaining species found within the site represent mostly species of secondary rainforests. However, floristic lists from a nearby CSIRO 0.5 ha plot at Oliver Creek (16°08'S, 145°26'E) reveal the presence of a number of different species (Graham 2006; Bradford et al. 2014), reflecting the compositional variability of lowland tropical rainforests in the Daintree region, presumably due to the very steep topography and local microclimate effects. A very conspicuous pattern that emerges for the DRO plots is the very low mean number of species per family (3.1). This is very low compared to other sites in Southeast Asia (e.g. Bukit Timah, Singapore: 5.31 species per family) and the Neotropics (e.g. Yasuni, Ecuador: 9.93 species per family) (Ostertag et al. 2014), and may be reflective of a higher rate of extinctions in the Australian tropics throughout past glacial-interglacial cycles (Byrne et al. 2011). This has perhaps also been added to by the immigration of selected Southeast Asian plant lineages over the last 20 million years (Sniderman and Jordan 2011; Crayn et al. 2014)

Nevertheless, the floristics of the 2 x 1-ha lowland rainforest plots reflect the high species endemism found within the Wet Tropics World Heritage Area tropical rainforest in Australia, with the prevalence of Lauraceae, Myrtaceae, Proteaceae and Sapindaceae typical of the region (e.g. Graham 2006; Bradford et al. 2014). The endemic tree *E. microneura* (Lauraceae), shrub *Haplostichanthus ramiflorus* (Annonaceae) and palm *Normanbya normanbyi* (Arecaceae) are not only locally abundant, but are the most abundant and highest basal area contributing species in their respective lifeform groups. There is also considerable overlap of families and genera and also a number of species with forests in South-East Asia. The families Lauraceae, Meliaceae, Myrtaceae, and Sapindaceae are well represented in some South-East Asian forests (Sist and Saridan 1999; Lee et al. 2002), although the family Dipterocarpaceae is conspicuously absent from the Wet Tropics. However, Gondwanan families (*sensu* Thorne 1986) less represented or absent from South-East Asian forest such as Atherospermataceae, Cunoniaceae, Eupomatiaceae, Monimiaceae and Proteaceae are fairly well represented in the DRO plots and in the region. The occurrence of these families align with the idea that these ancestral taxa occurred on the supercontinent before its breakup 55 million years ago (Sniderman and Jordan 2011).

**Figure 4.**
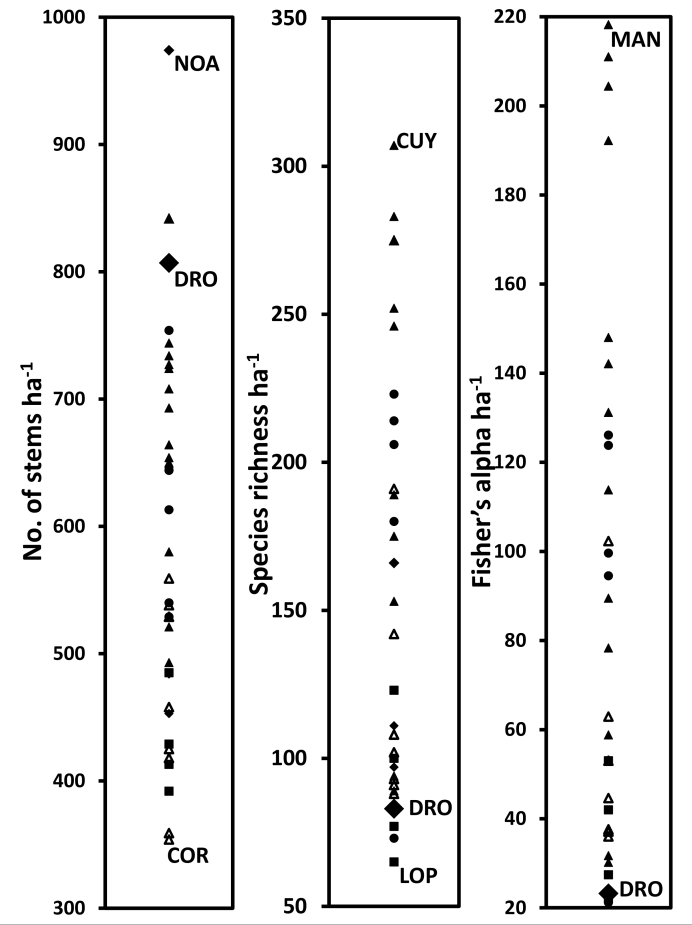
Relative ranking of the Daintree Rainforest Observatory (DRO) plots (1 ha averages) (large closed diamonds), Cape Tribulation, Australia in stem density (a), species richness (b), and Fisher’s alpha diversity indices (c), compared with selected tropical rainforest 1-ha monitoring plots in Australasia (including Papua New Guinea) (closed diamond) Southeast Asia (closed circles), Africa (squares), Central America (open triangles) and South America (closed triangles). Fisher’s alpha diversity takes into account the density of stem per species. For each graph, the highest and lowest sites are indicated (COR: Corcovado, Costa Rica; CUY: Cuyabeno, South America; GUN: Gunung Mulu, Southeast Asia; LOP: Lopé, Africa; MAN: Manaus, Brazil; NOA: Noah Creek, Australia). The data was compiled from Leigh (1999), Laidlaw et al. (2007) and Laurance et al. (2010).

Despite high endemism, the recorded species richness and Fisher’s alpha of tree stems ≥ 10 cm dbh is relatively low compared to Neotropical and South-East Asian plots of similar altitudes (Leigh 1999) (Fig. 4). However, there are sites from Palaeotropical plots that are lower in these indices than the DRO plots. Particularly noteworthy is the fact that the DRO core plot (807 stems ha^-1^) and similar plots in the region (e.g. Robson Creek - 937 stems ha^-1^: Bradford et al. 2014) have a much higher stem density than most Neotropical plots, which seldom exceeded 700 stems ha^-1^ (Fig. 4). Despite these high stem numbers, the tree basal area of 33.5 m^2^ ha^-1^ of the DRO plots are relatively typical of other rainforest plots in the Asian region (see Table 2 in Ostertag et al. 2014). These unusual patterns of high stem density and moderate basal area/biomass could be reflective of a dominance of mid-life cycle species adapted to disturbance caused by frequent tropical cyclones in the lowland rainforest as can be seen in the size class distribution where there are very few trees in the larger size classes (>70cm dbh) that are known to drive high biomass in moist lowland rainforests (Slik et al. 2013).

Indeed, cyclones play a strong role in shaping the structure and composition of tropical forests in the region (Webb 1958; Metcalfe et al. 2008; Murphy et al. 2013). For instance, the dominance of subcanopy species with the ability to form multiple stems such as *Cleistanthus myrianthus* and *Macaranga subdentata* can be an adaptive response to cyclones. Likewise, the very dense wood and other wood physical properties of dominant palms *Normanbya normanbyi* and *Licuala ramsayi* (both with wood densities exceeding 1 g cm^-3^: this study) may confer these species resistance to cyclones (e.g. Griffith et al. 2008), and explain their dominance in the plot. Moreover, the frequent occurrence of long-lived pioneer trees such as *Alstonia scholaris* and *Elaeocarpus angustifolius* also indicates past cyclone impacts. Most conspicuously, the presence of “vine towers”, formed by high cover of fast-growing lianas, particularly *Merremia peltata*, over trees, is a tell-tale sign of cyclone influence (Webb 1958; Metcalfe et al. 2008; Laurance and Curran 2008). The Daintree Rainforest Observatory has in its relatively short life-time (14 years) already been impacted by 3 cyclones, the first Cyclone Rona, causing massive disturbance to the crane plot (Turton and Siegenthaler 2004).

### Additional datasets

The current research aligns with a stimulus to enhance the research and teaching infrastructure of the facility, and we have built a reference herbarium of over 1200 specimens representing c.1000 species from the Daintree-Cape Tribulation region, and also voucher specimens from this study housed within the facility. Recently, a rainfall exclusion experiment has been set up under a 0.5 ha section under the crane plot to monitor tree and shrub responses to rainfall exclusion (Laurance 2015), along with a compilation of community plant functional trait data (e.g. Atkin 2012; Apgaua et al. 2015). In addition, ancillary faunal (Williams 2014, Williams 2015) and biophysical monitoring of litterfall (Edwards et al. in review), coarse woody debris, plant flowering phenology, soil and groundwater, and atmospheric flux measurements (http://www.ozflux.org.au/monitoringsites/capetribulation) are ongoing. Collectively, these multiple streams of data will enable us to closely monitor and experimentally understand the impacts of anthropogenically-induced climate change on lowland tropical rainforest.

## Supplementary Material

Supplementary material 1 Daintree Rainforest Observatory vascular plant species list and stem abundances (≥ 10 cm dbh) within the 2 x 1-ha monitoring plots. Data type: occurences, frequency
Brief description: Species within the Daintree Rainforest Observatory and stem abundances (≥ 10 cm dbh) within the 2 x 1-ha monitoring plots. The presence of nontree lifeforms are indicated with a (+).
File: oo_77730.docx Tng DYP, Apgaua DMGA, Campbell MJ, Cox CJ, Crayn D, Ishida FY, Laidlaw M, Liddell MJ, Seager M, Laurance SGW

## Figures and Tables

**Table 1.** Diversity and aboveground biomass measures of tree stems ≥ 10 cm diameter at breast height from the Daintree Rainforest Observatory 2 x 1-ha lowland rainforest plots. S = total number of species, n = number of stems, d = Margalef's species richness index, H' = Shannon diversity index, J' = Pielou's evenness index, E = Simpsons evenness index, BA = tree basal area (m^2^ ha^-1^).

	**S**	**N**	**D**	**H'**	**F**	**J'**	**E**	**BA**
Total plots (2 x 1-ha)	116	1531	15.83	3.779	29.25	0.792	0.959	66.8
Crane plot (1-ha)	85	698	12.61	3.608	24.29	0.812	0.951	33.3
Core plot (1-ha)	81	833	11.89	3.379	22.17	0.769	0.938	33.5

**Table 2.** The 10 most important families of tree stems ≥ 10 cm diameter at breast height from the Daintree Rainforest Observatory 2 x 1-ha lowland rainforest plots. Ni = number of individuals, Nsp = number of species, BA = basal area (m^2^), RDi = relative diversity, RF = relative frequency, RDo = relative dominance, FIV = family importance value.

**Family**	**Ni**	**Nsp**	**BA**	**RDi**	**RF**	**RDo**	**FIV**
Lauraceae	201	16	12.13	0.15	0.13	0.18	45.81
Arecaceae	284	3	4.43	0.03	0.19	0.07	27.74
Proteaceae	102	5	9.37	0.04	0.07	0.14	24.96
Myrtaceae	87	10	6.73	0.09	0.06	0.10	24.44
Meliaceae	59	10	4.04	0.08	0.04	0.06	17.45
Euphorbiaceae	155	3	2.99	0.03	0.10	0.04	17.16
Apocynaceae	91	5	3.78	0.05	0.06	0.06	16.91
Phyllanthaceae	149	2	3.18	0.02	0.10	0.05	16.20
Rutaceae	65	5	3.21	0.05	0.04	0.05	14.17
Myristicaceae	66	2	2.75	0.02	0.04	0.04	10.14

**Table 3.** Demographics of the 15 most abundant species (stems ≥ 10 cm dbh) within the 2 x 1-ha Daintree Rainforest Observatory lowland rainforest plot. D = stem density, F = number of 20 m x 20 m subplots present out of 50 subplots, BA = basal area (m^2^), RDe = relative density, RF = relative frequency, RDo = relative dominance, IVI = importance value index (Curtis 1959).

**Species**	**D**	**F**	**BA**	**RDe**	**RF**	**RDo**	**IVI**
*Cleistanthus myrianthus*	147	41	3.13	4.79	4.95	4.68	14.41
*Endiandra microneura*	69	32	4.91	2.25	3.86	7.34	13.45
*Cardwellia sublimis*	31	21	6.31	1.01	2.53	9.43	12.97
*Normanbya normanbyi*	147	16	2.35	4.79	1.93	3.52	10.24
*Alstonia scholaris*	63	25	2.03	2.05	3.02	3.04	8.11
*Macaranga subdentata*	108	12	2.05	3.52	1.45	3.06	8.03
*Myristica globosa*	63	16	2.65	2.05	1.93	3.96	7.94
*Licuala ramsayi*	123	2	1.81	4.01	0.24	2.71	6.96
*Cryptocarya mackinnoniana*	38	22	1.90	1.24	2.65	2.84	6.73
*Austromuellera trinervia*	45	25	1.46	1.47	3.02	2.21	6.69
*Argyrodendron peralatum*	24	17	2.02	0.78	2.05	3.01	5.85
*Castanospermum australe*	14	9	2.81	0.46	1.09	4.20	5.74
*Syzygium graveolens*	23	6	1.48	0.75	0.72	4.01	5.48
*Litsea leefeana*	15	35	0.45	0.49	4.22	0.67	5.38
*Antirhea tenuiflora*	34	27	0.44	1.11	3.26	0.66	5.03

**Table 4.** Demographics of tree species saplings (1-10cm dbh) within nine 20 m^2^ subplots within the Daintree Rainforest Observatory crane plot. D = stem density, % F = percentage of total tree sapling stems, % BA = percentage of total shrub basal area (m^2^).

**Species**	**D**	**% F**	**% BA**
*Endiandra microneura*	11	15.5	14.7
*Endiandra leptodendron*	3	4.2	13.2
*Medicosma fareana*	1	1.4	10.4
*Cleistanthus myrianthus*	16	22.5	9.6
*Brombya platynema*	2	2.8	8.6
*Siphonodon membranaceus*	6	8.5	8.0
*Rockinghamia angustifolia*	4	5.6	6.1
*Dysoxylum alliaceum*	2	2.8	5.3
*Beilschmiedia bancroftii*	1	1.4	4.8
*Myristica globosa*	3	4.2	4.6
*Endiandra microneura*	11	15.5	14.7
*Endiandra leptodendron*	3	4.2	13.2

**Table 5.** Demographics of shrub species within nine 20 m^2^ subplots within the Daintree Rainforest Observatory crane plot. D = stem density, % F = percentage of total shrub stems, % BA = percentage of total shrub basal area (m^2^).

**Species**	**D**	**% F**	**% BA**
*Haplostichanthus ramiflorus*	76	52.1	71.90
*Atractocarpus hirtus*	24	16.4	7.97
*Bowenia spectabilis*	22	15.1	3.06
*Cordyline cannifolia*	7	4.8	4.88
*Amaracarpus nematopodus*	4	2.7	5.18
*Ardisia brevipedata*	4	2.7	1.76
*Aglaia meridionalis*	1	0.7	0.49
*Breynia stipitata*	1	0.7	0.24
*Corymborkis veratrifolia*	1	0.7	0.11
*Harpullia rhyticarpa*	1	0.7	0.35
*Ixora biflora*	1	0.7	2.99
*Pittosporum rubiginosum*	1	0.7	1.06
