# Supplementary material for: Vegetation and floristics of a lowland tropical rainforest in northeast Australia
Source: Biodivers Data J. 2016 Feb 24;(4):e7599. doi: 10.3897/BDJ.4.e7599 (PMC4822074; doi:10.3897/BDJ.4.e7599)
Supplement: Supplementary material 1 — Daintree Rainforest Observatory vascular plant species list and stem abundances (≥ 10 cm dbh) within the 2 x 1-ha monitoring plots. [file biodiversity_data_journal-4-e7599-s001.docx]

**Table A1. Daintree Rainforest Observatory vascular plant species list and stem abundances (≥ 10 cm dbh) within the 2 x 1-ha monitoring plots. The presence of nontree lifeforms are indicated with a (+).**

| **Species** | **Family** | **Lifeform** | **Status (* = exotic,  E = endemic to Australia)** | **Plot presence (+)/ Abundance** |
| --- | --- | --- | --- | --- |
| **Species within the 2 x 1-ha plots** |  |  |  |  |
| *Adenanthera pavonina* | Fabaceae | Tree |  | + |
| *Aglaia meridionalis* | Meliaceae | Shrub | E | + |
| *Aglaia sapindina* | Meliaceae | Tree |  | + |
| *Aglaia tomentosa* | Meliaceae | Tree |  | + |
| *Alphitonia petriei* | Rhmanaceae | Tree | E | 1 |
| *Alpinia caerulea* | Zingiberaceae | Herb | E | + |
| *Alstonia muelleriana* | Apocynaceae | Tree |  | 4 |
| *Alstonia scholaris* | Apocynaceae | Tree |  | 63 |
| *Amaracarpus nematopodus* | Rubiaceae | Shrub | E | + |
| *Amyema conspicua* subsp. *conspicua* | Loranthaceae | Epiphyte |  | + |
| *Amyema quaternifolia* | Loranthaceae | Epiphyte | E | + |
| *Antirhea tenuiflora* | Rubiaceae | Tree |  | 34 |
| *Archidendron ramiflorum* | Fabaceae | Tree | E | 2 |
| *Archontophoenix alexandrae* | Arecaceae | Tree | E | 14 |
| *Ardisia brevipedata* | Primulaceae | Shrub | E | + |
| *Argyrodendron peralatum* | Malvaceae | Tree | E | 24 |
| *Arthropteris palisotii* | Tectariaceae | Hemiepiphyte |  | + |
| *Asplenium nidus* | Aspleniaceae | Epiphyte |  | + |
| *Atractocarpus hirtus* | Rubiaceae | Shrub | E | + |
| *Austromuellera trinervia* | Proteaceae | Tree | E | 45 |
| *Austrosteenisia blackii* | Fabaceae | Vine | E | + |
| *Balanophora fungosa* | Balanophoraceae | Herb |  | + |
| *Barringtonia calyptrata* | Lecythidaceae | Tree |  | 1 |
| *Beilschmiedia bancroftii* | Lauraceae | Tree | E | 14 |
| *Blechnum cartilagineum* | Blechnaceae | Herb | E | + |
| *Bowenia spectabilis* | Zamiaceae | Shrub | E | + |
| *Brachychiton acerifolius* | Malvaceae | Tree | E | 4 |
| *Brackenridgea austraVine* | Ochnaceae | Shrub | E | 1 |
| *Breynia stipitata* | Phyllanthaceae | Shrub |  | + |
| *Bridelia insulana* | Phyllanthaceae | Tree |  | + |
| *Brombya platynema* | Rutaceae | Tree | E | 29 |
| *Caesalpinia traceyi* | Fabaceae | Vine | E | + |
| *Calamus australis* | Arecaceae | Vine | E | + |
| *Calamus moti* | Arecaceae | Vine | E | + |
| *Calamus radicaulis* | Arecaceae | Vine | E | + |
| *Callicarpa longifolia* | Lamiaceae | Shrub |  | + |
| *Cananga odorata* | Annonaceae | Tree |  | 1 |
| *Canarium vitiense* | Burseraceae | Tree |  | + |
| *Carallia brachiata* | Rhizophoraceae | Tree |  | + |
| *Cardiopteris moluccana* | Cardiopteridaceae | Vine |  | + |
| *Cardwellia sublimis* | Proteaceae | Tree | E | 31 |
| *Carronia protensa* | Menispermaceae | Vine | E | + |
| *Casearia dallachii* | Salicaceae | Tree | E | + |
| *Castanospermum australe* | Fabaceae | Tree |  | 14 |
| *Castanospora alphandii* | Sapindaceae | Tree | E | + |
| *Cayratia saponaria* | Vitaceae | Vine | E | + |
| *Celtis paniculata* | Cannabaceae | Tree |  | 2 |
| *Cerbera floribunbda* | Apocynaceae | Tree |  | 7 |
| *Chisocheton longistipitatus* | Meliaceae | Tree |  | 1 |
| *Christella hispidula* | Thelypteridaceae | Herb |  | + |
| *Christella subpubescens* | Thelypteridaceae | Herb |  | + |
| *Cissus hastata* | Vitaceae | Vine |  | + |
| *Cissus penninervis* | Vitaceae | Vine |  | + |
| *Cissus vinosa* | Vitaceae | Vine | E | + |
| *Citronella smythii* | Icacinaceae | Tree | E | 3 |
| *Cleistanthus myrianthus* | Phyllanthaceae | Tree |  | 147 |
| *Clerodendrum tracyanum* | Lamiaceae | Shrub |  | + |
| *Colysis ampla* | Polypodiaceae | Hemiepiphyte | E | + |
| *Commersonia bartramia* | Malvaceae | Tree |  | 1 |
| *Cordyline cannifolia* | Asparagaceae | Shrub | E | + |
| *Corymborkis veratrifolia* | Orchidaceae | Shrub |  | + |
| *Crepidomanes barnardianum subsp. barnardianum* | Hymenophyllaceae | Epiphyte |  | + |
| *Crepidomanes bipunctatum var. bipunctatum* | Hymenophyllaceae | Epiphyte | E | + |
| *Crepidomanes saxifragoides* | Hymenophyllaceae | Epiphyte |  | + |
| *Cryptocarya cunninghamii* | Lauraceae | Tree |  | + |
| *Cryptocarya grandis* | Lauraceae | Tree | E | 12 |
| *Cryptocarya hypospodia* | Lauraceae | Tree |  | 3 |
| *Cryptocarya laevigata* | Lauraceae | Tree |  | + |
| *Cryptocarya mackinoniana* | Lauraceae | Tree |  | 38 |
| *Cryptocarya murrayi* | Lauraceae | Tree | E | 15 |
| *Cryptocarya oblata* | Lauraceae | Tree | E | + |
| *Cryptocarya pleurosperma* | Lauraceae | Tree | E | + |
| *Cryptocarya triplinervis* | Lauraceae | Tree | E | + |
| *Cupaniopsis diploglottoides* | Sapindaceae | Shrub | E | + |
| *Darlingia darlingiana* | Proteaceae | Tree | E | 2 |
| *Davidsonia pruriens* | Cunoniaceae | Shrub | E | 1 |
| *Decaspermum humile* | Myrtaceae | Tree | E | 7 |
| *Dendrocnide moroides* | Urticaceae | Shrub |  | + |
| *Dendrophthoe curvata* | Loranthaceae | Epiphyte |  | + |
| *Derris sp. Daintree (D.E.Boyland 469)* | Fabaceae | Vine | E | + |
| *Dichapetalum papuanum* | Dichapetalaceae | Vine |  | + |
| *Didymoglossum motleyi* | Hymenophyllaceae | Epiphyte |  | + |
| *Didymoglossum tahitense* | Hymenophyllaceae | Epiphyte |  | + |
| *Dioclea hexandra* | Fabaceae | Vine |  | + |
| *Diospyros cupulosa* | Ebenaceae | Tree |  | 1 |
| *Diospyros hebecarpa* | Ebenaceae | Tree |  | + |
| *Doryopteris concolor* | Dryopteridaceae | Epiphyte |  | + |
| *Doryphora aromatica* | Atherospermataceae | Tree | E | 4 |
| *Drynaria rigidula* | Polypodiaceae | Epiphyte |  | + |
| *Drypetes iodoformis* | Putranjivaceae | Tree | E | + |
| *Dysoxylum alliaceum* | Meliaceae | Tree |  | 11 |
| *Dysoxylum arborescens* | Meliaceae | Tree |  | 11 |
| *Dysoxylum mollissimum subsp. molle* | Meliaceae | Tree |  | 1 |
| *Dysoxylum oppositifolium* | Meliaceae | Tree |  | 4 |
| *Dysoxylum papuanum* | Meliaceae | Tree |  | 13 |
| *Dysoxylum parasiticum* | Meliaceae | Tree |  | 7 |
| *Dysoxylum pettigrewianum* | Meliaceae | Tree |  | 9 |
| *Elaeagnus triflora* | Elaeagnaceae | Vine |  | + |
| *Elaeocarpus angustifolius* | Elaeocarpaceae | Tree |  | 9 |
| *Elaeocarpus bancroftii* | Elaeocarpaceae | Tree | E | 9 |
| *Elaeocarpus grahamii* | Elaeocarpaceae | Tree | E | 5 |
| *Embelia caulialata* | Primulaceae | Vine |  | + |
| *Emmenospermum cunninghamii* | Rhamnaceae | Tree | E | 3 |
| *Endiandra acuminata* | Lauraceae | Tree | E | 5 |
| *Endiandra cowleyana* | Lauraceae | Tree | E | 1 |
| *Endiandra grayi* | Lauraceae | Tree | E | 1 |
| *Endiandra hypotephra* | Lauraceae | Tree | E | 5 |
| *Endiandra insignis* | Lauraceae | Tree | E | 1 |
| *Endiandra leptodendron* | Lauraceae | Tree | E | 14 |
| *Endiandra microneura* | Lauraceae | Tree | E | 69 |
| *Endiandra sankeyana* | Lauraceae | Tree | E | 1 |
| *Endiandra wolfei* | Lauraceae | Tree | E | 6 |
| *Entada phaseoloides* | Fabaceae | Vine |  | + |
| *Epipremnum pinnatum* | Araceae | Hemiepiphyte |  | + |
| *Erycibe coccinea* | Convolvulaceae | Vine | E | + |
| *Eupomatia laurina* | Eupomatiaceae | Shrub |  | 1 |
| *Eustrephus latifolius* | Asparagaceae | Vine |  | + |
| *Fagraea cambagei* | Gentianaceae | Tree |  | 3 |
| *Ficus congesta* | Moraceae | Tree |  | + |
| *Ficus copiosa* | Moraceae | Tree |  | 1 |
| *Ficus destruens* | Moraceae | Hemiepiphyte | E | + |
| *Ficus leptoclada* | Moraceae | Tree | E | + |
| *Ficus pantoniana* | Moraceae | Vine |  | + |
| *Ficus variegata* | Moraceae | Tree |  | 2 |
| *Ficus virgata* | Moraceae | Tree |  | + |
| *Flagellaria indica* | Flagellariaceae | Vine |  | + |
| *Flindersia bourjotiana* | Rutaceae | Tree | E | 2 |
| *Ganophyllum falcatum* | Sapindaceae | Tree |  | + |
| *Garcinia warrenii* | Clusiaceae | Tree |  | 8 |
| *Gardenia ovularis* | Rubiaceae | Tree | E | 2 |
| *Geophila repens* | Rubiaceae | Herb |  | + |
| *Gillbeea whypallana* | Cunoniaceae | Tree | E | 4 |
| *Glochidion sumatranum* | Phyllanthaceae | Tree |  | 2 |
| *Glossocarya hemiderma* | Lamiaceae | Vine |  | + |
| *Gmelina fasciculiflora* | Lamiaceae | Tree | E | 3 |
| *Gomphandra australiana* | Icacinaceae | Tree |  | 10 |
| *Grevillea baileyana* | Protaceae | Tree | E | 4 |
| *Guioa acutifolia* | Sapindaceae | Tree |  | 2 |
| *Gymnostachys anceps* | Araceae | Herb | E | + |
| *Haplostichanthus ramiflorus* | Annonaceae | Shrub | E | + |
| *Harpullia rhyticarpa* | Sapindaceae | Shrub | E | + |
| *Hedyotis auricularia var. melanesica* | Rubiaceae | Herb |  | + |
| *Hernandia albiflora* | Hernandiaceae | Shrub | E | + |
| *Hippocratea barbata* | Celastraceae | Vine | E | + |
| *Homalanthus novoguineensis* | Euphorbiaceae | Tree |  | + |
| *Homalium circumpinnatum* | Salicaceae | Tree | E | 1 |
| *Hornstedtia scottiana* | Zingiberaceae | Herb |  | + |
| *Hoya pottsii* | Apocynaceae | Vine |  | + |
| *Hypserpa decumbEs* | Menispermaceae | Vine | E | + |
| *Hypserpa laurina* | Menispermaceae | Vine |  | + |
| *Ichnanthus pallens* | Poaceae | Herb |  | + |
| *Ichnocarpus frutescens* | Apocynaceae | Vine |  | + |
| *Ipomoea velutina* | Convolvulaceae | Vine | E | + |
| *Ixora biflora* | Rubiaceae | Shrub | E | + |
| *Jasminum didymum subsp. didymum* | Oleaceae | Vine | E | + |
| *Kopsia arborea* | Apocynaceae | Tree |  | 3 |
| *Lasianthus kurzii* | Rubiaceae | Shrub |  | + |
| *Leea indica* | Vitaceae | Shrub |  | + |
| *Lepidozamia hopei* | Zamiaceae | Tree | E | 2 |
| *Licuala ramsayi var. ramsayi* | Arecaceae | Tree | E | 123 |
| *Lindsaea brachypoda* | Lindsaeaceae | Herb | E | + |
| *Linospadix minor* | Arecaceae | Shrub | E | + |
| *Litsea bindoniana* | Lauraceae | Tree | E | + |
| *Litsea leefeana* | Lauraceae | Tree | E | 15 |
| *Lygodium reticulatum* | Lygodiaceae | Vine |  | + |
| *Macaranga involucrata var. mallotoides* | Euphorbiaceae | Shrub | E | + |
| *Macaranga subdentata* | Euphorbiaceae | Tree | E | 108 |
| *Maesa dependens var. dependens* | Primulaceae | Vine | E | + |
| *Mallotus paniculatus* | Euphorbiaceae | Tree |  | 9 |
| *Marsdenia hemipteran* | Apocynaceae | Vine | E | + |
| *Medicosma fareana* | Rutaceae | Shrub | E | 18 |
| *Medicosma sessiliflora* | Rutaceae | Tree | E | + |
| *Melicope vitiflora* | Rutaceae | Tree | E | 15 |
| *Melicope xanthoxyloides* | Rutaceae | Tree | E | 1 |
| *Melodinus acutifolia* | Apocynaceae | Vine | E | + |
| *Melodinus australis* | Apocynaceae | Vine |  | + |
| *Merremia peltata* | Convolvulaceae | Vine |  | + |
| *Microlepia speluncae* | DEstaedtiaceae | Herb |  | + |
| *Mischocarpus grandissimus* | Sapindaceae | Tree | E | 1 |
| *Mucuna gigantea* | Fabaceae | Vine |  | + |
| *Musgravea heterophylla* | Proteaceae | Tree | E | 20 |
| *Myristica globosa subsp. muelleri* | Myristicaceae | Tree | E | 63 |
| *Myristica insipida* | Myristicaceae | Tree |  | 3 |
| *Myrsine porosa* | Primulaceae | Tree | E | 2 |
| *Neolitsea dealbata* | Lauraceae | Tree | E | 1 |
| *Neonauclea glabra* | Rubiaceae | Tree |  | 1 |
| *Neosepicaea jucunda* | Bignoniaceae | Vine | E | + |
| *Nephrolepis acutifolia* | Nephrolepidaceae | Epiphyte |  | + |
| *Nephrolepis hirsutula* | Nephrolepidaceae | Herb |  | + |
| *Niemeyera prunifera* | Sapotaceae | Tree | E | 18 |
| *Normanbya normanbyi* | Arecaceae | Tree | E | 147 |
| *Oplismenus compositus* | Poaceae | Herb |  | + |
| *Ormosia ormondii* | Fabaceae | Tree | E | 1 |
| *Palaquium galactoxylum* | Sapotaceae | Tree |  | 7 |
| *Palmeria scandens* | Monimiaceae | Vine |  | + |
| *Pandorea pandorana* | Bignoniaceae | Vine |  | + |
| *Parapachygone longifolia* | Menispermaceae | Vine | E | + |
| *Pararistolochia deltantha* | Aristolochiaceae | Vine | E | + |
| *Parsonsia latifolia* | Apocynaceae | Vine | E | + |
| *Parsonsia longipetiolata* | Apocynaceae | Vine | E | + |
| *Parsonsia velutina* | Apocynaceae | Vine |  | + |
| *Passiflora kuranda* | Passifloraceae | Vine | E | + |
| *Phlegmariurus phlegmaria* | Lycopodiaceae | Epiphyte |  | + |
| *Pilidiostigma papuanum* | Myrtaceae | Shrub |  | + |
| *Piper caninum* | Piperaceae | Vine |  | + |
| *Piper hederaceum var. hederaceum* | Piperaceae | Vine | E | + |
| *Pittosporum rubiginosum* | Pittosporaceae | Shrub | E | + |
| *Pityrogramma calomelanos* | Pteridaceae | Herb | * | + |
| *Planchonella chartacea* | Sapotaceae | Tree |  | 6 |
| *Planchonella myrsinodendron* | Sapotaceae | Tree |  | 2 |
| *Platycerium hillii* | Polypodiaceae | Epiphyte | E | + |
| *Polyscias australiana* | Araliaceae | Tree |  | 8 |
| *Polyscias elegans* | Araliaceae | Tree |  | 1 |
| *Pothos longipes* | Araceae | Hemiepiphyte |  | + |
| *Pouteria xerocarpa* | Sapotaceae | Tree | E | 1 |
| *Premna serratifolia* | Lamiaceae | Shrub |  | 1 |
| *Prunus turneriana* | Rosaceae | Tree |  | 3 |
| *Pseuderanthemum variabile* | Acanthaceae | Herb |  | + |
| *Pseuduvaria froggattii* | Annonaceae | Tree | E | + |
| *Psilotum nudum* | Psilotaceae | Epiphyte |  | + |
| *Psychotria dallachiana* | Rubiaceae | Shrub | E | + |
| *Psydrax tropica* | Rubiaceae | Tree | E | 4 |
| *Pycnarrhena novoguineensis* | Menispermaceae | Vine |  | + |
| *Pyrrosia longifolia* | Polypodiaceae | Epiphyte |  | + |
| *Rhaphidophora australasica* | Araceae | Hemiepiphyte | E | + |
| *Rhaphidophora hayi* | Araceae | Hemiepiphyte |  | + |
| *Rhodamnia sessiliflora* | Myrtaceae | Tree | E | 1 |
| *Rockinghamia angustifolia* | Euphorbiaceae | Tree | E | 38 |
| *Rourea brachyandra* | Connaraceae | Vine |  | + |
| *Rubus moluccanus var. moluccanus* | Rosaceae | Vine |  | + |
| *Ryparosa kurrangii* | Achariaceae | Tree |  | + |
| *Salacia disepala* | Celastraceae | Vine | E | + |
| *Sarcopteryx reticulata* | Sapindaceae | Tree | E | + |
| *Schefflera actinophylla* | Araliaceae | Tree |  | + |
| *Schizaea dichotoma* | Schizaeaceae | Herb |  | + |
| *Semecarpus australiensis* | Anacardiaceae | Tree |  | 2 |
| *Siphonodon membranaceus* | Celastraceae | Tree | E | 2 |
| *Sloanea langii* | Elaeocarpaceae | Tree | E | 7 |
| *Smilax australis* | Smilacaceae | Vine | E | + |
| *Steganthera laxiflora spp. laxiflora* | Monimiaceae | Tree | E | 2 |
| *Sterculia quadrifida* | Malvaceae | Tree |  | 1 |
| *Strychnos minor* | Loganiaceae | Vine |  | + |
| *Symplocos paucistaminea* | Symplocaceae | Shrub |  | 1 |
| *Synima cordierorum* | Sapindaceae | Tree |  | 9 |
| *Syzygium claviflorum* | Myrtaceae | Tree |  | 3 |
| *Syzygium cormiflorum* | Myrtaceae | Tree | E | 6 |
| *Syzygium graveolens* | Myrtaceae | Tree | E | 23 |
| *Syzygium gustavioides* | Myrtaceae | Tree | E | 8 |
| *Syzygium hemilamprum subsp. hemilamprum* | Myrtaceae | Tree |  | 3 |
| *Syzygium kuranda* | Myrtaceae | Tree | E | 11 |
| *Syzygium monospermum* | Myrtaceae | Tree |  | 20 |
| *Syzygium sayeri* | Myrtaceae | Tree |  | 6 |
| *Tabernaemontana pandacaqui* | Apocynaceae | Shrub |  | + |
| *Tarenna dallachiana subsp. dallachiana* | Rubiaceae | Shrub |  | + |
| *Ternstroemia cherry* | Pentaphyllaceae | Shrub |  | + |
| *Tetracera daemeliana* | Dilleniaceae | Vine | E | + |
| *Tetracera nordtiana* | Dilleniaceae | Vine |  | + |
| *Tetrastigma nitens* | Vitaceae | Vine | E | + |
| *Toechima erythrocarpum* | Sapindaceae | Tree |  | 6 |
| *Toona ciliata* | Meliaceae | Tree |  | 1 |
| *Trophis scandens subsp. scandens* | Moraceae | Vine |  | + |
| *Uvaria uhrii* | Annonaceae | Vine | E | + |
| *Ventilago ecorollata* | Rhamnaceae | Vine | E | + |
| *Vitex queenslandica* | Lamiaceae | Tree | E | 3 |
| *Vittaria elongate* | Vittariaceae | Epiphyte |  | + |
| *Wrightia laevis* | Apocynaceae | Tree |  | 14 |
| *Xanthophyllum octandrum* | Polygalaceae | Tree | E | 25 |
| *Zeuxine oblonga* | Orchidaceae | Herb | E | + |
| **Additional species recorded from the DRO** |  |  |  |  |
| *Acacia celsa* | Fabaceae | Tree | E |  |
| *Acacia crassicarpa* | Fabaceae | Tree |  |  |
| *Acacia flavescens* | Fabaceae | Tree | E |  |
| *Acacia mangium* | Fabaceae | Tree |  |  |
| *Acronychia acronychioides* | Rutaceae | Tree | E |  |
| *Adenia heterophylla subsp. heterophylla* | Passifloraceae | Vine |  |  |
| *Alphitonia oblata* | Rhmanaceae | Tree |  |  |
| *Alphitonia whitei* | Rhamnaceae | Tree | E |  |
| *Amphineuron terminans* | Thelypteridaceae | Herb |  |  |
| *Antidesma erostre* | Phyllanthaceae | Tree | E |  |
| *Antrophyum callifolium* | Vittariaceae | Epiphyte |  |  |
| *Archidendron lucyi* | Fabaceae | Tree |  |  |
| *Artocarpus heterophyllus* | Moraceae | Tree | * |  |
| *Arytera pauciflora* | Sapindaceae | Tree | E |  |
| *Atractocarpus fitzalanii* subsp*.fitzalanii* | Rubiaceae | Shrub | E |  |
| *Atractocarpus sessilis* | Rubiaceae | Shrub | E |  |
| *Austrosteenisia stipularis* | Fabaceae | Vine | E |  |
| *Axonopus compressus* | Poaceae | Herb | * |  |
| *Beilschmiedia obtusifolia* | Lauraceae | Tree |  |  |
| *Benstonea monticola* | Pandanaceae | Shrub | E |  |
| *Bulbostylis barbata* | Cyperaceae | Herb |  |  |
| *Calophyllum australianum* | Clusiaceae | Tree | E |  |
| *Calopogonium mucunoides* | Fabaceae | Vine | * |  |
| *Cayratia japonica* | Vitaceae | Vine |  |  |
| *Centella asiatica* | Apiaceae | Herb |  |  |
| *Centotheca lappacea* | Poaceae | Herb |  |  |
| *Chamaecrista rotundifolia* | Fabaceae | Herb | * |  |
| *Chionanthus ramiflorus* | Oleaceae | Tree |  |  |
| *Chrysopogon aciculatus* | Poaceae | Herb | * |  |
| *Cleistanthus discolor* | Phyllanthaceae | Tree | E |  |
| *Cleome aculeata* | Capparidaceae | Herb | * |  |
| *Commersonia macrostipulata* | Malvaceae | Tree | E |  |
| *Costus potierae* | Costaceae | Herb | E |  |
| *Crassocephalum crepidioides* | Asteraceae | Herb | * |  |
| *Crepidomanes humile* | Hymenophyllaceae | Epiphyte |  |  |
| *Crotalaria lanceolata* | Fabaceae | Shrub | * |  |
| *Cyanthillium cinereum* | Asteraceae | Herb |  |  |
| *Cyperus aromaticus* | Cyperaceae | Herb |  |  |
| *Cyperus metzii* | Cyperaceae | Herb | * |  |
| *Cyperus sphacelatus* | Cyperaceae | Herb |  |  |
| *Cyrtococcum oxyphyllum* | Poaceae | Herb |  |  |
| *Dactyloctenium aegyptium* | Poaceae | Herb | * |  |
| *Deplanchea tetraphylla* | Bignoniaceae | Tree |  |  |
| *Derris trifoliata* | Fabaceae | Vine |  |  |
| *Desmodium heterocarpon* | Fabaceae | Herb |  |  |
| *Dianella bambusifolia* | Hemerocallidaceae | Herb |  |  |
| *Digitaria ciliaris* | Poaceae | Herb | * |  |
| *Dillenia alata* | Dilleniaceae | Tree |  |  |
| *Diplocyclos palmatus* | Cucurbitaceae | Vine |  |  |
| *Diploglottis bernieana* | Sapindaceae | Tree | E |  |
| *Drymaria cordata* | Caryophyllaceae | Herb | * |  |
| *Dysoxylum gaudichaudianum* | Meliaceae | Tree |  |  |
| *Dysoxylum rufum* | Meliaceae | Tree |  |  |
| *Echinochloa colona* | Poaceae | Herb | * |  |
| *Elaeocarpus culminicola* | Elaeocarpaceae | Tree |  |  |
| *Eleusine indica* | Poaceae | Herb | * |  |
| *Endiandra anthropophagorum* | Lauraceae | Tree | E |  |
| *Endospermum myrmecophilum* | Euphorbiaceae | Tree |  |  |
| *Euphorbia hirta* | Euphorbiaceae | Herb | * |  |
| *Eupomatia barbata* | Eupomatiaceae | Shrub | E |  |
| *Euroschinus falcata* | Anacardiaceae | Tree | E |  |
| *Ficus benjamina* | Moraceae | Tree |  |  |
| *Ficus crassipes* | Moraceae | Hemiepiphyte | E |  |
| *Ficus drupacea* | Moraceae | Tree |  |  |
| *Ficus hispida* | Moraceae | Tree |  |  |
| *Ficus septica* | Moraceae | Tree |  |  |
| *Ficus virens* | Moraceae | Tree |  |  |
| *Fimbristylis dichotoma* | Cyperaceae | Herb |  |  |
| *Freycinetia scandens* | Pandanaceae | Hemiepiphyte |  |  |
| *Glochidion harveyanum var. harveyanum* | Phyllanthaceae | Tree |  |  |
| *Glochidion philippicum* | Phyllanthaceae | Tree |  |  |
| *Goniophlebium percussum* | Polypodiaceae | Epiphyte |  |  |
| *Helicia australasica* | Proteaceae | Shrub |  |  |
| *Helicia nortoniana* | Proteaceae | Shrub | E |  |
| *Heliconia sp.* | Heliconiaceae | Herb | * |  |
| *Hibiscus tiliaceus* | Malvaceae | Tree |  |  |
| *Hydriastele wendlandiana* | Arecaceae | Tree | E |  |
| *Hyptis capitata* | Lamiaceae | Shrub | * |  |
| *Hyptis pectinata* | Lamiaceae | Shrub | * |  |
| *Ilex arnhemensis* | Aquifoliaceae | Tree |  |  |
| *Intsia bijuga* | Fabaceae | Tree |  |  |
| *Ipomoea indica* | Convolvulaceae | Vine | * |  |
| *Ipomoea triloba* | Convolvulaceae | Vine |  |  |
| *Jasminum elongatum* | Oleaceae | Vine |  |  |
| *Lantana camara* | Verbenaceae | Shrub | * |  |
| *Legnephora moorei* | Menispermaceae | Vine | E |  |
| *Lindernia antipoda* | Linderniaceae | Herb |  |  |
| *Lindernia crustacea* | Linderniaceae | Herb |  |  |
| *Litsea fawcettiana* | Lauraceae | Tree | E |  |
| *Lophostemon suaveolens* | Myrtaceae | Tree |  |  |
| *Ludwigia hyssopifolia* | Onagraceae | Shrub | * |  |
| *Macaranga tanarius* | Euphorbiaceae | Tree |  |  |
| *Mallotus mollissimus* | Euphorbiaceae | Shrub |  |  |
| *Mangifera indica* | Anacardiaceae | Tree | * |  |
| *Mecardonia procumbens* | Scrophulariaceae | Herb | * |  |
| *Megathyrsus maximus* | Poaceae | Herb | * |  |
| *Melastoma malabathricum* | Melastomataceae | Shrub |  |  |
| *Melia azedarach* | Meliaceae | Tree |  |  |
| *Melicope bonwickii* | Rutaceae | Tree |  |  |
| *Melicope elleryana* | Rutaceae | Tree |  |  |
| *Melinis repens* | Poaceae | Herb | * |  |
| *Merremia quinquefolia* | Convolvulaceae | Vine | * |  |
| *Micromelum minutum* | Rutaceae | Tree |  |  |
| *Mimosa pudica* | Fabaceae | Herb | * |  |
| *Mischocarpus exangulatus* | Sapindaceae | Tree | E |  |
| *Molineria capitulata* | Hypoxidaceae | Herb |  |  |
| *Mollugo pentaphylla* | Molluginaceae | Herb | * |  |
| *Odontonema tubaeforme* | Acanthaceae | Shrub | * |  |
| *Oldenlandia corymbosa* | Rubiaceae | Herb | * |  |
| *Ophioglossum pendulum* | Ophioglossaceae | Epiphyte |  |  |
| *Oplismenu hirtellus* | Poaceae | Herb |  |  |
| *Oplismenu undulatifolius var. mollus* | Poaceae | Herb |  |  |
| *Ottochloa nodosa* | Poaceae | Herb |  |  |
| *Paspalum conjugatum* | Poaceae | Herb | * |  |
| *Passiflora foetida* | Passifloraceae | Vine | * |  |
| *Phlegmariurus phlegmarioides* | Lycopodiaceae | Epiphyte |  |  |
| *Phyllanthus debilis* | Phyllanthaceae | Herb | * |  |
| *Phyllanthus novae-hollandiae* | Phyllanthaceae | Vine |  |  |
| *Physalis minima* | Solanaceae | Herb | * |  |
| *Pipturus argenteus* | Urticaceae | Shrub |  |  |
| *Pittosporum ferrugineum* | Pittosporaceae | Shrub |  |  |
| *Pittosporum trilobum* | Pittosporaceae | Shrub | E |  |
| *Planchonella obovata* | Sapotaceae | Tree |  |  |
| *Polygala paniculata* | Polygalaceae | Herb | * |  |
| *Polyscias murrayi* | Araliaceae | Tree | E |  |
| *Pouteria browlessiana* | Sapotaceae | Tree | E |  |
| *Praxelis clematidea* | Asteraceae | Herb | * |  |
| *Psychotria loniceroides* | Rubiaceae | Shrub | E |  |
| *Ptisana oreades* | Marattiaceae | Herb | E |  |
| *Pueraria phaseoloides* | Fabaceae | Vine | * |  |
| *Randia audasii* | Rubiaceae | Shrub | E |  |
| *Rhus taitensis* | Anacardiaceae | Shrub |  |  |
| *Rhysotoechia robertsonii* | Sapindaceae | Tree | E |  |
| *Richardia brasiliensis* | Rubiaceae | Herb | * |  |
| *Salacia erythrocarpa* | Celastraceae | Vine |  |  |
| *Sarcopetalum harveyanum* | Menispermaceae | Vine | E |  |
| *Scolopia braunii* | Salicaceae | Tree | E |  |
| *Scoparia dulcis* | Scrophulariaceae | Herb | * |  |
| *Selaginella longipinna* | Selaginellaceae | Herb | E |  |
| *Senna obtusifolia* | Fabaceae | Shrub | * |  |
| *Sida rhombifolia* | Malvaceae | Shrub | * |  |
| *Smilax calophylla* | Smilacaceae | Vine |  |  |
| *Smilax sp. aff. S. elliptica* | Smilacaceae | Vine | ? |  |
| *Solanum nigrum* | Solanaceae | Herb | * |  |
| *Solanum torvum* | Solanaceae | Shrub | * |  |
| *Sonchus oleraceus* | Asteraceae | Herb | * |  |
| *Spermacoce latifolia* | Rubiaceae | Herb | * |  |
| *Sphagneticola trilobata* | Asteraceae | Herb | * |  |
| *Stachytarpheta cayensis* | Verbenaceae | Shrub | * |  |
| *Stylosanthes humilis* | Fabaceae | Shrub | * |  |
| *Synedrella nodiflora* | Asteraceae | Herb | * |  |
| *Syngonium podophyllum* | Araceae | Hemiepiphyte | * |  |
| *Syzygium angophoroides* | Myrtaceae | Tree | E |  |
| *Syzygium australe* | Myrtaceae | Tree | E |  |
| *Syzygium fibrosum* | Myrtaceae | Tree |  |  |
| *Syzygium luehmannii* | Myrtaceae | Tree | E |  |
| *Syzygium xerampelinum* | Myrtaceae | Tree | E |  |
| *Terminalia catappa* | Combretaceae | Tree |  |  |
| *Terminalia sericocarpa* | Combretaceae | Tree | E |  |
| *Terminalia subacroptera* | Combretaceae | Tree |  |  |
| *Tetrastigma thornsborneorum* | Vitaceae | Vine | E |  |
| *Timonius timon var. timon* | Rubiaceae | Shrub |  |  |
| *Trema cannabina* | Cannabaceae | Shrub |  |  |
| *Trema orientalis* | Cannabaceae | Tree |  |  |
| *Trema tomentosa* | Cannabaceae | Shrub |  |  |
| *Tridax procumbens* | Asteraceae | Herb | * |  |
| *Tristaniopsis exiliflora* | Myrtaceae | Tree | E |  |
| *Uncaria lanosa var. appendiculata* | Rubiaceae | Vine |  |  |
| *Urochloa decumbens* | Poaceae | Herb | * |  |
| *Urochloa mutica* | Poaceae | Herb | * |  |
| *Xanthostemon chrysanthus* | Myrtaceae | Tree | E |  |
